# Local Construction Employment and Binge Drinking Among US Adults in Their Early 30s: Insights From a Path Analysis

**DOI:** 10.1002/ajim.70119

**Published:** 2026-07-29

**Authors:** Sehun Oh, Bridget Freisthler, Devan Hawkins

**Affiliations:** ^1^ College of Social Work, The Ohio State University Columbus Ohio USA; ^2^ College of Social Work, The University of Tennessee Knoxville Tennessee USA; ^3^ Public Health Program, School of Arts and Sciences, Massachusetts College of Pharmacy and Health Sciences Boston Massachusetts USA

**Keywords:** binge drinking, construction, path analysis, psychological distress, United States

## Abstract

**Objective:**

Construction workers face elevated risks of alcohol misuse—especially binge drinking—due to physically demanding labor, long hours, and limited paid vacation. At the same time, emerging evidence suggests that a larger local construction sector may have protective community‐level effects on alcohol and other drug outcomes. This study examines how both county‐level construction job share and individual employment in construction relate to residents’ binge‐drinking behaviors, incorporating individual and work‐related mediators.

**Methods:**

Using data from the National Longitudinal Survey of Youth 1997, linked with county‐level job statistics, we applied generalized structural equation modeling to assess associations between local construction job share and binge drinking among adults in their early 30s. Mediators included employment status, work schedule, precarious employment conditions, and psychological distress.

**Results:**

Living in counties with a larger construction sector increased the likelihood of working in frontline construction occupations, which was associated with more demanding work schedules. Demanding work schedule and psychological distress were positively associated with binge drinking. However, employment in construction also predicted lower precarious employment conditions and psychological distress. Notably, greater local construction job share was directly associated with lower odds and frequency of binge drinking, suggesting a protective ecological effect.

**Conclusions:**

Findings highlight the importance of addressing demanding work hours and limited vacation—key risk factors for alcohol misuse. Although a strong construction economy may offer community‐level protective effects, targeted interventions remain essential to mitigate alcohol‐related risks within this high‐risk occupational group.

## 1 Introduction

Construction workers in the United States face a disproportionately high risk of alcohol misuse and related adverse outcomes. Shockey and Esse [1] report that 26.3% of individuals in construction occupations reported binge drinking, the highest rate among all occupation groups. Alarmingly, 21% of construction workers in their 30s consume alcohol before or during work, raising concerns about workplace safety and existing substance use policies [2]. Prior research attributes elevated alcohol misuse in this sector to several interrelated factors, including emotional stress from physically‐demanding labor and long hours, chronic pain that may encourage self‐medication, and permissive drinking norms—particularly in male‐dominated environments [3, 4, 5].

Construction employment is also associated with higher wages (average weekly earnings of $1510 in 2024 vs. $1209 across private sectors) and relatively stable employment, particularly for workers without a college degree [6, 7]. These features may reduce precarious employment and psychological distress. Although construction‐specific evidence remains limited, broader research links employment stability and higher earnings to lower psychological distress [8, 9] and reduced alcohol misuse [10, 11]. Together, construction work may involve both risk‐elevating (e.g., demanding schedules, physical strain) and risk‐reducing (e.g., financial stability) pathways.

At the ecological level, counties with larger construction sectors exhibit lower rates of substance‐related risks [12, 13]. Although these findings do not directly reflect individual behavior, they suggest that local economic conditions may influence population health. Construction‐oriented economies are often associated with lower unemployment, greater job availability across related industries (e.g., supply chain, logistics, and services), and increased government revenues and infrastructure investments [6, 14]. This is consistent with the multiplier‐effect literature documenting that periods of heightened construction employment coincide with local economic expansion [15]. It also aligns with evidence indicating that stable employment opportunities can be protective against substance‐related harms, particularly among individuals with low income and fewer skills [16]. While these findings support research linking local economic conditions to substance‐use behaviors [17], less is known about whether specific alcohol consumption patterns, particularly binge drinking, vary across different local economic contexts, representing an important gap in the literature.

These observations highlight the need to examine how both risk‐elevating and risk‐reducing pathways may shape binge drinking among construction workers and how local construction job share relates to community‐level health outcomes. Prior studies have largely examined these processes separately, focusing on either individual‐level occupational factors or ecological associations with local economic conditions, with limited integration within a single analytic framework using US population‐based data. Because the construction sector may entail both risk‐elevating working conditions and risk‐reducing economic benefits, examining work‐related and psychological pathways is essential to understanding these multilevel patterns. To address this gap, we use longitudinal data to estimate a path model linking local construction job share and employment in frontline construction occupations to binge drinking among young workers, incorporating work‐related and psychological mediators. This study provides an integrated assessment of individual‐ and community‐level pathways linking construction employment to alcohol‐related risk.

## 2 Methods

### 2.1 Data and Sample

Data were drawn from the 2009–2011 waves of the National Longitudinal Survey of Youth 1997 (NLSY97), linked with the Longitudinal Employer‐Household Dynamics Origin‐Destination Employment Statistics (LODES). NLSY97 is a nationally representative longitudinal study of individuals born between 1980 and 1984 with data on education, employment, and health behaviors, including binge drinking. US Census Bureau's LODES data include job counts by work and worker characteristics, including two‐digit North American Industry Classification System (NAICS) industry codes at the census‐block level. Industry‐specific job counts were aggregated to the county level and linked to NLSY97 respondents based on county of residence. Of the 6842 NLSY97 respondents who participated in 2009–2011 waves (76.2% retention), the analytic sample included 6604 after excluding 238 respondents (3.5%) with missing county of residence or binge drinking data.

### 2.2 Measures

Binge drinking: The primary outcome is past‐month binge drinking (measured in 2011), defined as consuming five or more drinks on a single occasion. As a supplemental measure, we examined binge‐drinking frequency, operationalized as the number of binge‐drinking days in the past month.

Construction job share and employment: Key independent variables included: (1) the county‐level proportion of jobs in the construction sector (NAICS Code 23) in 2009, and (2) individual‐level employment in frontline construction occupations in 2010, identified using the US Census Standard Occupational Classification codes 6200–6760 (e.g., carpenters, electricians, painters, roofers). In the United States, construction employment is typically embedded within local labor markets rather than employer‐provided residential compounds. Most construction workers live in surrounding communities and commute to job sites, and over 93% in our data are employees rather than self‐employed (Table A1).

Mediators: Three mediators measured in 2010 reflected employment conditions and psychological well‐being dimensions: (1) demanding work schedule, measured as a binary indicator (0 = *no*, 1 =* yes*) of working 40 h a week for 52 weeks while receiving fewer than 11 paid vacation days, that is, the mean number of paid vacation days among private industry employees after one year of service [18]. A supplemental measure captured highly‐demanding work schedule as working 55+ hours per week for 52 weeks based on the US Government Accountability Office's [19] definition with fewer than 11 paid vacation days; (2) precarious employment was measured as the mean of four indicators capturing temporariness, low wages, and uncertain working hours—adapted from Padrosa and colleagues [20], including: (a) non‐regular daytime work arrangements; (b) no earned income in the past year; (c) underemployment—working fewer than 15 weeks in the past year following the US Bureau of Labor Statistics’ U‐1 definition; and (d) low annual wages (earning less than $15,080 [full‐time earnings at the 2010 federal minimum wage, $7.25 per hour]), with a reliability coefficient of *α* = 0.656; and (3) psychological distress: the mean score from the five‐item short version of the Mental Health Inventory [21]. Respondents rated how often they experienced specific emotional states over the past month on a four‐point scale (1 = *all of the time* to 4 = *none of the time*), including feeling nervous, calm/peaceful, downhearted and blue, happy, and so down in the dumps that nothing could cheer them up. Positive items were reverse‐coded so that higher values indicated greater distress (*α* = 0.800).

### 2.3 Analytic Strategy

We estimated generalized structural equation models (GSEM) to examine associations between county‐level construction job share and binge drinking, with mediation through employment and psychological well‐being characteristics. GSEM extends traditional SEM to accommodate mixed measurement levels, non‐normal distributions, and non‐linear relationships [22, 23, 24]. Model 1 included direct paths from construction job share to binge‐drinking outcomes and proximal employment‐related mediators: individual employment in frontline construction occupations, and demanding work schedules. Model 2 added two mediators—precarious employment conditions and psychological distress—to capture broader employment strain and psychological well‐being. We then replicated the models using the number of binge‐drinking days in the past month to capture binge drinking frequency. All path model outcomes were adjusted for individual‐level controls to address potential confounding. We applied longitudinal weights from the NLSY97's custom weighting program to account for the survey's sampling design and attrition. Additional details on the NLSY97 are available at https://www.bls.gov/nls/nlsy97.htm. The study protocol (#2021B0089) was approved by the Institutional Review Board at The Ohio State University, the corresponding author's institution.

## 3 Results

Table A1 summarizes descriptive statistics of the analytic sample by construction employment status. Among the sample, 284 individuals (4.6%) were employed in construction. Compared with other respondents, construction workers were more likely to be male, have an associate's degree or less, work regular daytime hours, report longer annual work hours, receive fewer paid vacation days, and had higher prevalence and frequency of binge drinking.

**Figure 1:**
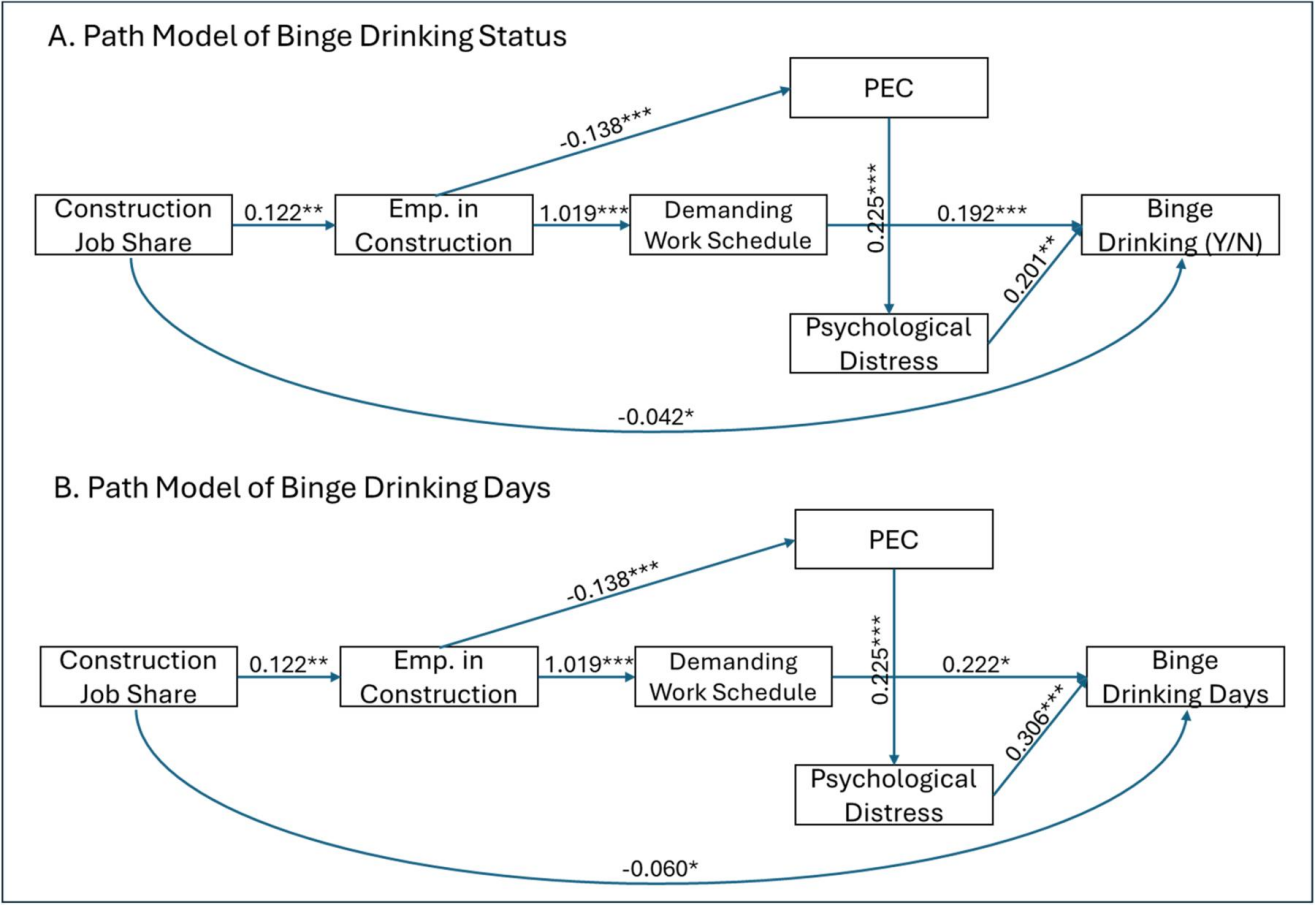
Path model diagram linking job shares in the construction sector to binge drinking: Generalized path analysis from the National Longitudinal Survey of Youth 1997 (*n* = 6,604). PEC, precarious employment conditions. Path coefficients were derived from the full models (Model 2) in Table 1 for both outcome measures. *p < 0.05, **p < 0.01, ***p < 0.001.

Table 1 presents path coefficients for both the occurrence and frequency. Higher county‐level construction job share was significantly associated with increased likelihood of individual employment in frontline construction occupations (*b* = 0.122, *p* < 0.01; AOR = 1.130, 95% CI = 1.046–1.221). Employment in construction predicted higher demanding work schedule (*b* = 1.019, *p* < 0.001; AOR = 2.771, 95% CI = 2.132–3.603). In the full models (Model 2; Figure 1), employment in construction was associated with lower precarious employment conditions (*b* = −0.138, *p* < 0.001). Precarious employment conditions significantly predicted greater psychological distress (*b* = 0.225, *p* < 0.001). Direct paths to binge drinking revealed that higher county‐level construction job share was associated with lower odds of binge drinking (*b* = −0.042, *p* < 0.05; AOR = 0.959, 95% CI = 0.922–0.998). In contrast, demanding work schedule (*b* = 0.192, *p* < 0.001, [AOR = 1.212, 95% CI = 1.042–1.410]) and psychological distress (*b* = 0.201, *p* < 0.01 [AOR = 1.222, 95% CI = 1.068–1.399]) were positively associated with binge drinking. Similar patterns emerged for binge‐drinking frequency.

**Table 1:** Path coefficients linking job shares in the construction sector to binge drinking: Generalized path analysis from the National Longitudinal Survey of Youth 1997 (*n* = 6604).

Path			*b*	se	exp(*b*)	95% CI
*Common paths*						
Construction Job Share	→	Emp in Construction	0.122**	0.039	1.130	1.046–1.221
Emp in Construction	→	Demanding Work Schedule	1.019***	0.133	2.771	2.132–3.603
*Paths to outcome 1: Binge drinking status in the past month (Y/N)*						
Model 1: Base model						
Construction Job Share	→	Binge Drinking (Y/N)	−0.043*	0.020	0.958	0.920–0.997
Demanding Work Schedule			0.187*	0.075	1.206	1.039–1.400
Emp in Construction			0.164	0.128	1.178	0.914–1.519
Model 2: Full model						
Emp in Construction	→	Precarious Emp. Conditions	−0.138***	0.019		
Precarious Emp. Conditions	→	Psychological Distress	0.225***	0.018		
Construction Job Share	→	Binge Drinking (Y/N)	−0.042*	0.020	0.959	0.922–0.998
Demanding Work Schedule			0.192***	0.076	1.212	1.042–1.410
Psychological Distress			0.201**	0.068	1.222	1.068–1.399
*Paths to outcome 2: Number of binge drinking days in the past month*						
Model 1: Base model						
Construction Job Share	→	Binge Drinking (No. of Days)	0.062*	0.024	0.943	0.896–0.987
Demanding Work Schedule			0.219*	0.081	1.245	1.060–1.463
Emp in Construction			0.170	0.123	1.185	0.928–1.513
Model 2: Full model						
Emp in Construction	→	Precarious Emp. Conditions	−0.138***	0.019		
Precarious Emp. Conditions	→	Psychological Distress	0.225***	0.018		
Construction Job Share	→	Binge Drinking (No. of Days)	−0.060*	0.024	0.942	0.898–0.988
Demanding Work Schedule			0.222*	0.080	1.249	1.066–1.462
Psychological Distress			0.306***	0.077	1.358	1.166–1.583

*Note:* Outcome variables were controlled for sex, race/ethnicity, highest degrees received, and marital status in 2011. In Model 2, the non‐significant path from employment in construction to binge drinking (identified in Model 1) was excluded. An additional model (not shown) tested the path from demanding work schedules to psychological distress, which was not statistically significant. This suggests that the relationship between demanding work schedules and binge drinking may not be mediated by psychological distress. *p < 0.05; **p < 0.01; ***p < 0.001.

Table A2 presents results using highly‐demanding rather than demanding work schedule. Employment in construction significantly increased highly‐demanding work schedule (*b* = 0.768, *p* < 0.001; AOR = 2.156, 95% CI = 1.435–3.240), although the effect size was smaller than that observed for demanding work schedule. Notably, highly‐demanding work schedules were associated with greater increases in both occurrence and frequency than demanding work schedule.

## 4 Discussion

Our findings suggest a complex relationship between construction employment and binge drinking. Consistent with international evidence linking long working hours to increased risky alcohol consumption among young workers [25], construction employment is associated with demanding work schedule—long hours and limited paid vacation—which may encourage binge drinking as a form of stress relief from physically demanding labor [26]. However, this pathway did not appear to operate through psychological distress, as demanding schedules were not significantly linked to psychological distress in our model, highlighting the difficulty of attributing binge drinking directly to work conditions alone. At the same time, employment in construction was associated with reduced precarious employment conditions, which were linked to lower psychological distress and reduced binge‐drinking risk, consistent with prior evidence [8, 9, 10, 11]. Although we could not compare the strength of these opposing pathways, prior research showing elevated binge drinking among construction workers suggests that the risks tied to long hours and limited vacation may outweigh the protective effects of stable employment and higher compensation [1].

Our ecological findings further illustrate the dual role of the local construction sector in shaping binge‐drinking patterns among residents. A higher concentration of construction jobs increased the likelihood of employment in frontline construction occupations, which—through mechanisms described above—may elevate binge drinking risks among workers. We also observed a protective ecological effect: counties with a larger construction job share exhibited lower binge drinking risk. This supports evidence that a strong construction sector may confer broader community‐level benefits [12, 13], potentially through expanded employment in supply chain and service sectors, income growth, and improved public infrastructure [6, 14]. Nonetheless, the elevated risk among construction workers underscores the need for targeted interventions addressing occupational burden through managing long work hours and ensuring adequate paid vacation.

Several limitations should be noted. First, our model did not separately estimate various workplace‐specific factors that may shape alcohol misuse, nor could it fully disentangle construction‐specific labor‐market effects from contemporaneous macroeconomic conditions that often accompany construction upswings. The protective effects of construction‐job share may reflect improved economic conditions that may reduce drinking behavior. In addition, the data do not capture the timing, location, or social context of binge drinking, limiting our ability to directly attribute binge drinking to occupational exposure. Future research is needed to identify the mechanisms through which a robust construction sector may exert protective effects, while isolating broader macroeconomic influences. Second, the data are 13 years old and may not reflect the current state of the construction industry or its workforce. For instance, the growing representation of Black workers over the past decade may have introduced shifts in alcohol‐related behaviors and workplace dynamics due to lower alcohol use behavior among Black Americans and other racial/ethnic groups [27]. Third, the data set predates the newer binge drinking criteria that differentiate thresholds for men and women (the 5/4 split). Lastly, our study does not distinguish among construction occupations, socio‐professional positions, or firm size despite substantial heterogeneity in wage and working conditions. Future research should explore these differences using larger and more diverse samples.

Despite these limitations, this study contributes to the literature on occupational health and alcohol misuse by integrating individual‐level employment conditions with local construction job concentration within a single analytic framework. In doing so, it helps reconcile elevated binge‐drinking risk among construction workers with protective associations observed at the community level. The findings underscore the importance of mitigating the impact of demanding work hours and insufficient vacation—both recognized risk factors for alcohol misuse. While a strong construction economy may offer protective benefits at the community level, targeted, occupation‐specific interventions remain essential to reduce alcohol misuse among construction workers facing demanding work schedules and limited vacation time.

## Author Contributions

**Sehun Oh:** conceptualization, methodology, data curation, formal analysis, writing – original draft, visualization, funding acquisition. **Bridget Freisthler:** conceptualization, methodology, writing – review and editing, supervision. **Devan Hawkins:** conceptualization, methodology, writing – original draft.

## Disclosure

The authors have nothing to report.

## Ethics Statement

The study protocol (#2021B0089) was approved by the Institutional Review Board at The Ohio State University, where the study was conducted.

## Conflicts of Interest

The authors declare no conflicts of interest.

## Data Access

This research was conducted with restricted access to Bureau of Labor Statistics (BLS) data. The views expressed here do not necessarily reflect the views of the BLS.

## Data Availability

The data used in this study are derived from the restricted‐use National Longitudinal Survey of Youth 1997 (NLSY97). These data are not publicly available; access is governed by a data use agreement with the US Bureau of Labor Statistics (BLS) and requires formal approval to ensure the confidentiality of respondents.

## Appendix A

**Table A1:** Sample characteristics for the total sample and by construction employment status.

	Total (*n* = 6604)		Employed in construction jobs			
			Yes (*n* = 284; 4.6%)		No (*n* = 6320; 95.4%)	
	Mean	95% CI	Mean	95% CI	Mean	95% CI
**Sociodemographics**						
Male (%)	49.37	47.88–50.87	97.01	94.52–98.38	47.09	45.61–48.58
Race/ethnicity (%)						
Black	15.93	14.21–17.80	10.14	7.04–14.39	16.20	14.46–18.11
Hispanic	12.77	11.46–14.20	14.11	10.15–19.29	12.71	11.43–14.11
Multiracial	1.32	0.96–1.81	0.47	0.01–3.26	1.36	0.98–1.89
White	69.98	67.84–72.04	75.28	69.61–80.19	69.73	67.58–71.79
Educational attainment (%)						
High school/equivalent	7.68	6.85–8.61	14.36	10.34–19.59	7.36	6.55–8.26
Associate/junior college	53.29	51.66–54.92	75.38	69.00–80.81	52.22	50.57–53.86
Bachelor's degree	7.75	6.92–8.66	4.18	2.18–7.86	7.92	7.07–8.86
Graduate degree	31.28	29.56–33.05	6.08	3.86–9.46	32.51	30.77–34.29
Marital status in 2011 (%)						
Never married	50.92	49.29–52.55	52.91	46.25–59.47	50.83	49.23–52.42
Married	40.66	39.35–41.99	39.98	33.24–47.11	40.70	39.35–42.06
S/D/W	8.41	7.67–9.23	7.11	4.36–11.38	8.48	7.72–9.30
IPR in 2011 (%)						
0–99	12.56	11.51–13.70	12.91	8.23–19.69	12.54	11.47–13.71
100–199	19.41	18.23–20.66	22.59	17.69–28.38	19.26	18.05–20.53
200+	68.02	66.26–69.73	64.50	57.86–70.62	68.20	66.39–69.95
**Employment characteristics (2010)**						
Non‐regular day work (%)	46.56	45.03–48.11	24.10	18.78–30.35	47.59	46.00–49.18
Unemployment (%)	17.18	15.89–18.56	9.13	6.26–13.12	17.57	16.21–19.02
Underemployment (%)	28.50	27.07–29.98	19.67	15.04–25.30	28.92	27.47–30.41
Self‐employment status (%)	3.21	2.75–3.74	6.71	3.81–11.54	3.04	2.57–3.59
Low wage (%)	19.83	18.63–19.52	13.38	8.95–19.52	20.16	18.93–21.46
Precarious employment conditions (Range: 0–1)	0.31	0.30–0.32	0.18	0.15–0.22	0.32	0.31–0.33
Annual work hours	1,613	1,583–1,643	1,945	1,858–2,033	1,597	1,568–1,628
Among those who worked at all	1,894	1,871–1,916	1,963	1,884–2,041	1,890	1,867–1,912
Days of paid vacations	5.87	5.64–6.09	2.61	1.97–3.25	6.03	5.80–6.26
Among those who worked	6.86	6.60–7.11	2.63	1.99–3.27	7.10	6.83–7.37
Demanding work schedule (%)	28.77	27.69–29.87	51.45	45.20–57.67	27.70	26.59–28.85
Highly‐demanding work schedule (%)	6.38	5.70–7.14	12.31	8.66–17.20	6.10	5.43–6.85
**Behavioral health**						
Psychological distress in 2010	1.87	1.85–1.88	1.73	1.67–1.78	1.87	1.86–1.89
Binge drinking in 2011						
Prevalence (%)	33.94	32.28–35.64	44.59	39.13–50.18	33.43	31.73–35.17
Number of days in past month	1.45	1.34–1.57	2.45	1.84–3.07	1.40	1.30–1.51

*Notes:* IPR = Income‐to‐Poverty Threshold Ratio. Unemployment is defined as having received no income in the past year; underemployment is defined as having worked fewer than 15 weeks in the past year, based on U.S. Bureau of Labor Statistics’ U1 definition; Low wage is defined as earning less than the annual income one would receive working full time (40 hours/week for 52 weeks) at the 2010 minimum wage of $7.25; Demanding work schedule is defined as working 2,080 hours in the past year (i.e., 40+ hours a week for 52 weeks) while receiving fewer vacation days than the private industry average of 11 days. Reliability scores for precarious employment conditions and psychological distress (MHI‐5) items are 0.6564 and 0.7994, respectively.

**Table A2:** Path coefficients linking job shares in the construction sector to binge drinking using highly‐demanding work schedule: Generalized path analysis from the national longitudinal survey of youth 1997 (*n* = 6,604).

Path			*b*	se	Exp(b)	95% CI
**Common paths**						
Construction Job Share	→	Emp in Construction	0.122**	0.039	1.130	1.046–1.221
Emp in Construction	→	Highly‐demanding work schedule	0.768***	0.201	2.156	1.435–3.240
**Paths to outcome 1: Binge drinking status in the past month (Y/N)**						
*Model 1: Base Model*						
Construction Job Share	→	Binge Drinking (Y/N)	−0.042*	0.020	0.959	0.921–0.998
Highly‐demanding work schedule			0.323**	0.121	1.381	1.086–1.756
Emp in construction			0.188	0.131	1.207	0.932–1.564
*Model 2: Full Model*						
Emp in Construction	→	Precarious Emp. Conditions	−0.138***	0.019		
Precarious Emp. Conditions	→	Psychological Distress	0.225***	0.018		
Construction Job Share	→	Binge Drinking (Y/N)	−0.041*	0.020	0.960	0.922–0.999
Highly‐Demanding Work Schedule			0.324***	0.122	1.383	1.086–1.761
Psychological Distress			0.197**	0.069	1.217	1.061–1.396
**Paths to Outcome 2: Number of binge drinking days in the past month**						
*Model 1: Base Model*						
Construction job share	→	Binge Drinking (No. of Days)	−0.059*	0.024	0.943	0.898–0.990
Highly‐demanding work schedule			0.307*	0.127	1.359	1.057–1.748
Emp in construction			0.192	0.125	1.211	0.946–1.552
*Model 2: Full Model*						
Emp in construction	→	Precarious Emp. Conditions	−0.138***	0.019		
Precarious Emp. Conditions	→	Psychological Distress	0.225***	0.018		
Construction Job Share	→	Binge Drinking (No. of Days)	−0.057*	0.024	0.945	0.900–0.992
Highly‐Demanding Work Schedule			0.277**	0.122	1.320	1.036–1.682
Psychological Distress			0.296***	0.076	1.344	1.157–1.562

*Notes:* Outcome variables were controlled for sex, race/ethnicity, highest degrees received, and marital status in 2011. In Model 2, the non‐significant path from employment in construction to binge drinking (identified in Model 1) was excluded. An additional model (not shown) tested a path from highly‐demanding work schedule to psychological distress. The path was not statistically significant, suggesting that the relationship between highly‐demanding work schedule and binge drinking may not be mediated by psychological distress.
